# Insulin resistance in hypothyroid patients under Levothyroxine therapy: a comparison between those with and without thyroid autoimmunity

**DOI:** 10.1186/s40200-014-0103-4

**Published:** 2014-10-30

**Authors:** Tina Mazaheri, Faranak Sharifi, Koorosh Kamali

**Affiliations:** Zanjan University of Medical Sciences, Zanjan, Iran; Metabolic Diseases Research Center, Zanjan University of Medical Sciences, Zanjan, Iran; Department of public health, Zanjan University of Medical Sciences, Zanjan, Iran

**Keywords:** Hypothyroidism, Autoimmunity, Insulin resistance

## Abstract

**Background:**

A chronic inflammation resulting from an imbalance between pro-inflammatory and anti-inflammatory cytokines in Hashimoto’s thyroiditis (HT) might be responsible for IR in hypothyroidism. This study was performed to investigate a probable association between autoimmune background of hypothyroidism and IR.

**Methods:**

In this clinical study, 63 subjects with Hashimoto’s thyroiditis and 49 subjects with post-ablation hypothyroidism were enrolled. All the participants were euthyroid for more than one year through Levothyroxine therapy. Serum concentrations of Thyroid-stimulating Hormone (TSH), Free Thyroxin (FT4, FT3), Anti-Thyroid Peroxidase Antibodies (Anti-TPO Abs), Total Cholesterol (TC), HDL-Cholesterol (HDL-C), Triglyceride (TG), Fasting Blood Glucose (FBG), and insulin levels were measured and Oral Glucose Tolerance Test (OGTT) was performed for all of the subjects. Participants with anti TPO levels more than 1000 IU /ml were classified as having highly positive antibodies.

**Results:**

No significant differences regarding to plasma insulin, glucose and lipid concentration, were detected between subjects with and without Hashimoto’s thyroiditis. However, subjects with highly positive Anti TPO Abs had higher prevalence of elevated fasting insulin level than those with lower titers of Anti TPO Abs and subjects without autoimmune background (94.1% *vs.* 62.8% and 71.4% respectively, P = 0.05). Subjects with highly positive titers of Abs also had a lower serum HDL-c levels than the rest of the subjects (40.6 ± 2.1 vs. 47.2 ± 1.7 and 47.4 ± 1.4, P = 0.04).

**Conclusions:**

There is no obvious association between thyroid autoimmunity and metabolic indexes of hypothyroid patients. Only patients with Ani TPO antibody levels more than 1000 IU/ml may experience higher insulin level and less HDL-c with the same BMI.

## Introduction

Hypothyroidism has been reported in 1% to 10% of the adult population [1] and is accompanied by a number of important health implications including increased risk of dyslipidemia, altered peripheral glucose disposal due to insulin resistance and cardiovascular disorders [2,3]. Insulin resistance is a key factor in the pathogenesis of type 2 diabetes mellitus (DM), metabolic syndrome and atherosclerosis [1,4]. It is established that hypothyroidism constitutes to insulin resistant state [5,6]. Furthermore, some studies have shown that even a subtle increase in plasma TSH levels within the physiological range may affect insulin secretion [7] and may be associated with insulin resistance and metabolic syndrome [8]. However, the exact mechanism connecting hypothyroidism to IR is still unclear. The fact that overt hypothyroidism and even subclinical hypothyroidism have been associated with disorders of glucose and insulin metabolism [9,10] and that thyroid replacement therapy has been unable to restore Insulin Mediated Glucose Uptake (IMGU) to its physiological state suggests that molecular and cellular interactions other than the thyroid hormones are involved in the insulin resistance state [11].

Since Hashimoto’s thyroiditis is the most common cause of hypothyroidism in areas with sufficient iodine intake [12], a chronic inflammation resulting from an imbalance between pro-inflammatory and anti-inflammatory cytokines [13] in this disease might be the mechanism responsible for IR in hypothyroidism. Considering these theory non-autoimmune and non-inflammatory causes of hypothyroidism would not contribute to IR.

To illustrate a probable association between autoimmune background of hypothyroidism and IR this study was conducted. In this study we compared insulin resistance and dysglycemia in hypothyroid patients with and without autoimmunity after attainment of a euthyroid state through levothyroxine therapy.

## Materials and methods

### Subjects

A total of 112 adult hypothyroid patients including 63 subjects with Hashimoto’s thyroiditis and 49 people with post ablation hypothyroidism who were randomly selected from Valiasr Hospital outpatient endocrine clinic, an academic general hospital in Zanjan, enrolled in this study. Diagnosis of hypothyroidism was conducted if serum TSH concentration was more than 5 IU/ml, and serum T4 concentration was less than 64 · 5 nmol/l. Individuals with serum TSH level more than 10 IU/ml accompanied by normal T4 and T3 levels, were considered as subclinical hypothyroid. All hypothyroid patients with enlarged rubbery thyroid and high serum Anti-TPO Abs concentration were categorized as Hashimoto’s thyroiditis while those with history of thyroid surgery or radioiodine ablation performed in more than a year period of time and without any anti-thyroid antibodies in their blood were recognized as post ablation hypothyroid.

Having a list of all hypothyroid patients of the clinic, they were categorized under Hashimoto’s thyroiditis and post-ablation hypothyroidism based on their documented medical history. Inclusion criteria were hypothyroid participants who underwent Levothyroxine therapy in an appropriate dosage by which the patient was euthyroid at least for one year. Patients with diabetes mellitus, cardiovascular disease, cerebral vascular disease, corticosteroid consumption, pregnancy, chronic liver disease, thyroid cancer, renal dysfunction and any autoimmune disease like lupus erythematous and Rheumatoid arthritis were excluded from the study. None of the selected individuals were under medication known to affect glucose or lipid metabolism.

At the beginning of the study anti-thyroid antibodies including anti thyroglobulin and Anti TPO levels were measured again for all the eligible participants and those with positive anti thyroid antibodies in the post ablation hypothyroid group were excluded from the study.

After exclusion of two subjects due to DM in Hashimoto’s thyroiditis group and 16 subjects from post-ablation hypothyroid group including 6 people with mild elevated Anti TPO Abs, four people with history of thyroid cancer, five subjects who did not complete the study and one person with DM, 112 patients were studied.

All the participants were informed orally about the aim of study and informed consents were obtained from them. This study was approved by the ethical committee of Zanjan Univesity of Medical Sciences in Iran.

### Measurements

Clinical examination and Anthropometric Measurements carried out for all the patients by the researcher. Height was measured with a stadiometer to the nearest 0.5 cm. Body weight was recorded by calibrated digital scale while wearing light clothes. Blood pressure values were measured twice on the left upper arm; in the sitting position with a random zero sphygmomanometer after a 10-min rest. Waist circumference between the iliac crest and the lowest rib at umbilicus level was measured using flexible tape. Body Mass Index (BMI) was also calculated accordingly. Demographic information and family history of diabetes and hypertension were obtained from the participants. Duration of replacement therapy with Levothyroxine-sodium, plasma TSH levels in the first diagnosis of hypothyroidism state and TSH plasma levels at the time of study were explored and documented specifically. In order to select only euthyroid patients, those with TSH or FT3 values below or above the normal range were excluded from further analysis.

Blood samples were collected from the ante-cubital vein of the participants after at least 12 hours of fasting. Serum concentrations of TSH, FT4, FT3, Anti TPO Abs, Total Cholesterol (TC), HDL-cholesterol (HDL-C), Triglyceride (TG), glucose and insulin were measured. Afterwards, an Oral Glucose Tolerance Test (OGTT) with 75 gr of glucose was performed for all the participants.

Obesity was defined as BMI ≥30 kg/m^2^, central obesity as a waist circumference ≥102 cm in men and ≥88 cm in women, low HDL as serum HDL cholesterol ≤40 mg/dl in men and ≤50 mg/dl in women. Serum triglycerides ≥150 mg/dl, total cholesterol level ≥240 mg/dl, fasting insulin >6 uIU/mL and fasting plasma glucose ≥100 mg/ dl were considered as hypertriglyceridemia, hypercholesterolemia, elevated fasting insulin level and hyperglycemia respectively. Hypertension defined as systolic blood pressure more than 130/85 mmHg. TPO Ab level of more than 80 U/ml was positive. People with anti TPO levels more than 1000 IU /ml were categorized as having highly positive antibodies and analyzed separately.

### Laboratory measurements

Biochemical variables measurements were done at the laboratory of Vali-e-asr Hospital, using commercial kits: Plasma glucose was measured by the glucose peroxidase colorimetric enzymatic method, with a sensivity of 5 mg/dl. Serum cholesterol and triglyceride (TG) were measured by colorimetric method with a sensivity of 5 mg/dl. Low-density lipoprotein (LDL-C) estimation was calculated using the Friedewald formula. Insulin resistance was estimably assessed by the homeostasis model assessment index (HOMA-IR = (fasting glucose (mmol/l) × fasting insulin (uIU/mL)/22.5).TSH concentrations were measured by Immuno Chemiluminescense assay. Anti-TPO was determined by radioimmunoassay (RIA) system (SorinBiomedia, Italy).

### Statistics

Statistical analyses were done using SPSS 16.5 software package. Data are expressed as mean ± SE or medium where is appropriate. Differences between two groups were tested using Student’s unpaired *t-*test (or Mann–Whitney according to sample distribution)_._ For an analytic comparison of the variables between three groups with and without a normal distribution, Fisher test and Kruskal-Wallis test, were utilized respectively. ϰ 2 test was performed to assess the significance of differences between proportions. To determine the correlation between the serum anti TPO antibody concentration and other variables Regression analysis was employed. P < 0.05 was considered to specify statistical significance.

## Results

A total of 112 hypothyroid patients including 98 (88%) women and 14 (12%) men with the mean age of 41.8 ± 12.8 years were studied. Basal clinical and laboratory characteristics of the two groups of patients with Hashimoto’s thyroiditis and post-ablation hypothyroidism are compared in Table 1. Based on the data, no significant difference regarding to clinical and biochemical variables was found between the hypothyroid patients with and without autoimmune background. Although serum levels of TG and HOMA-IR were relatively higher in patients with Hashimoto’s thyroiditis, the difference was not statistically significant.

**Table 1:** **Comparison of the biochemical and anthropometric parameters in two groups of patients based on their background autoimmunity (serum levels of Anti-TPO Abs)**

**Parameter**	**Post ablative hypothyroid (n:49)**	**Hashimoto’s thyroiditis (n:63)**	***P*** **value**
Age (year)	43.9 ± 1.7	39.9 ± 1.5	0.103
W.C (cm)	89.5 ± 3.1	92.6 ± 1.4	0.696
BMI (kg/m^2^)	28.5 ± 0.6	28.1 ± 0.6	0.741
SBP	113.3 ± 3.1	114.6 ± 2.2	0.677
DBP	72.2 ± 2	76.1 ± 1.8	0.126
TC (mmol/L)	189.2 ± 6	179.1 ± 5.6	0.266
TG (mmol/L)	134.4 ± 7.3	147.9 ± 11.6	0.935
HDL (mmol/L)	47.4 ± 1.4	45.6 ± 1.4	0.232
FPG (mmol/L)	93 ± 1.6	90.5 ± 1.7	0.452
OGTT	112.9 ± 3.7	108.2 ± 4.5	0.181
Insulin (uIU/mL)	8.9 ± 0.6	10.7 ± 1.1	0.615
HOMA-IR	2.1 ± 0.2	2.4 ± 0.3	0.913

*WC*: Waist Circumference; *BMI*: Body Mass Index; *SBP*: Systolic Blood Pressure; *DSB*: Diastolic Blood Pressure; *TC*: Total cholesterol; *TG*: Triglyceride; *HDL*: High Density Lipoprotein; *FPG*: Fasting Plasma Glucose, *OGTT*: Oral Glucose Tolerance Test.

Regarding to HOMA-IR, 50 subjects (44.6%) of hypothyroid patients were insulin resistant including 26 (41.2%) patients with Hashimoto’s thyroiditis and 24 (48.9%) patients with post ablation hypothyroidism (*P*: 0.232).

Patients with Anti TPO antibody levels more than 1000 IU/ml were classified as having highly positive antibodies, and were separately analyzed. Table 2 shows the difference between the three groups of patients for frequency of obesity, central obesity, hypercholesterolemia, hypertriglyceridemia, low-HDL cholesterol and hyperglycemia. The prevalence of hyperinsulinemia was significantly higher in Hashimoto patients with higher levels of Anti TPO antibodies more than 1000 IU/ml (Table 2).

**Table 2:** **Difference between the two groups of patients for frequency of obesity, central obesity, hyperchlosterolemia, hypertriglyceridemia, low-HDL cholesterol and hyperglycemia**

**Parameters**	**Post ablative hypothyroid (n:49)**	**Hashimoto’s thyroiditis (n:46)**	**Hashimoto’s thyroiditis with highly positive TPO abs (n:17)**	***P*** **value**
Obesity	20(40.8%)	14(30.4%)	5(31.3%)	0.539
Central obesity	37(75.5%)	28(60.9%)	11(64.7%)	0.301
Hypertension	6(12.2%)	6(13%)	3(17.6%)	0.851
Hypercholesterolemia	6(12.2%)	5(10.9%)	1(5.9%)	0.767
Hypertriglyceridemia	17(34.7%)	19(41.3%)	4(28.6%)	0.639
Low-HDL cholesterol	15(30.6%)	14(30.4 %)	8(47.1%)	0.414
Hyperglycemia	13(26.5%)	13(28.3%)	4(23.5%)	0.931
Elevated fasting insulin level	36(71.4%)	27(62.8%)	16(94.1%)	0.05

Table 3 illustrates an overview of the biochemical and anthropometric parameters in the group with highly positive anti thyroid antibodies and compares it with other hypothyroid patients. The data reveals that beside HDL-c which was significantly lower in patients with high levels of anti thyroid antibody, other parameters like insulin resistance were not different between the three groups.

**Table 3:** **Comparison of the biochemical and anthropometric parameters in three groups of patients based on their serum levels of Anti-TPO Abs**

**Paremeters**	**Negative Anti-TPO Abs N: 49**	**Positive Anti-TPO Abs N: 46**	**Highly positive Anti-TPO Abs N: 17**	**P value**
Age (year)	43.9 ± 1.8	40.9 ± 1.7	37.2 ± 3.2	0.13
W.C (cm)	89.5 ± 3.2	91.6 ± 0.7	96.2 ± 3.1	0.58
BMI (kg/m^2^)	28.5 ± 0.6	27.7 ± 0.7	30 ± 1.2	0.41
SBP	113.2 ± 3.1	113.9 ± 2.6	117.1 ± 5.8	0.91
DBP	72.1 ± 2	74.5 ± 2.2	78 ± 2.6	0.33
TC (mmol/L)	189.2 ± 6	179.9 ± 6.5	176.9 ± 11.7	0.46
TG (mmol/L)	134.4 ± 7.4	148.8 ± 13.6	145.2 ± 24.4	0.94
HDL (mmol/L)	47.4 ± 1.4	47.2 ± 1.7	40.6 ± 1.2	0.04
FPG (mmol/L)	93 ± 1.6	90.8 ± 2.1	89.6 ± 2.9	0.66
OGTT	112.9 ± 3.7	113.3 ± 5.4	92.9 ± 6.4	0.09
Insulin (uIU/mL)	8.9 ± 0.6	10.54 ± 1.5	11.07 ± 1	0.20
HOMA-IR	2.1 ± 0.2	2.4 ± 0.4	2.5 ± 0.3	0.29

Table 4 refers to the relationship between the serum level of Anti TPO antibody and other variables.

**Table 4:** **Relationship between the serum Anti TPO antibody concentration and other variables**

**Independent variable**	**Dependent variable**	***P*** **value**
	W.C (cm)	0.203
	BMI (kg/m^2^)	0.658
	SBP	0.833
	DBP	0.364
	TC (mmol/L)	0.253
Serum Anti TPO Abs level	TG (mmol/L)	0.821
	HDL (mmol/L)	0.091
	FPG (mmol/L)	0.701
	OGTT	0.078
	Insulin (uIU/mL)	0.880
	HOMA-IR	0.888

The mean duration of hypothyroidism in our patients was 5.5 ± 7.9 years. Participants were categorized into two groups based on Levothyroxine therapy duration. Glucose concentration and insulin resistance index of subjects who had been diagnosed with hypothyroidism and had received treatment within the past 5 years does not significantly differ from subjects who had been diagnosed earlier and undergone levothyroxine therapy for more than 5 years.

Patients were divided into three groups based on their current TSH levels while taking Levothyroxine for treatment. Patients with serum TSH levels of below 1.88, 1.88 to 3.40, and 3.41 to 5 showed no significant difference in terms of insulin resistance and hyperglycemia. Also no meaningful difference in terms of the presence of metabolic syndrome was detected among the groups (P = 0.39).

## Discussion

The results of current study revealed a high prevalence of insulin resistance in hypothyroid patients whereas they were in euthyroid state for more than one year with Levothyroxine therapy (44.8%). Moreover, the prevalence of elevated fasting insulin level was more significant in those with highly positive Anti TPO antibodies (94.1%). However; no significant differences were found regarding plasma Insulin, glucose and lipid concentration between patients with and without Hashimoto’s thyroiditis diagnosed as the cause of their hypothyroidism.

In recent years, tremendous interest has been raised in the effect of thyroid function on insulin levels. It is established that clinical hypothyroidism is considered as an insulin-resistant state [5,6]. Following their studies on patients with hypothyroidism, Handisurya [14] and Stanicka [15] have concluded that hypothyroidism makes glucose inaccessible to insulin [16]. However; an exact pathogenetic mechanism involved in insulin resistance in hypothyroidism is still unknown. In current study with enrolling different groups of hypothyroid patients, we tried to detect any association of autoimmunity against thyroid and insulin resistance. Adjusting for age and sex, hypothyroid patients had more frequency of central obesity than general population of Zanjan, the city that this study was conducted (67% *vs*. 40%) [17]. Having high prevalence of insulin resistance (44%) in our patients, who all were euthyroid for a long time before the study, reveals another mechanism other than the role of low thyroid hormones for the insulin resistance in hypothyroidism. Higher central fat in the subjects may explain the higher prevalence of insulin resistance among participants, however; a significantly more prevalence of elevated fasting insulin level was detected in a subgroup of patients with Hashimoto’s thyroiditis and highly elevated levels of Anti TPO antibodies more than 1000 IU/ml which might support the concept of autoimmunity role in insulin resistance. Regarding the metabolic indexes and insulin resistance, no differences were found between hypothyroid patients with and without autoimmunity against thyroid.

Positive effect of Levothyroxine on insulin resistance is still under investigation. Robert Krysiak et al. [18] have illustrated that a six-month treatment with levothyroxine does not have a significant effect on HOMA-IR in patients with hypothyroidism. Our results could support this finding as although we have not considered the level of glucose, lipid and insulin levels of our hypothyroid patients before the initiation of treatment, they had yet much more frequency of insulin resistance after a long time of treatment as compared to the general population [19]. Thus, a significant effect of replacement therapy with Levothyroxine on insulin metabolism in hypothyroid patients seems to be unlikely.

Whether the treatment with Levothyroxine can affect autoimmune background against thyroid or not is not clear. Based on some studies [18,20] treatment with Levothyroxine may attenuate the level of Anti TPO antibody in hypothyroid patients and patients whose hypothyroidism is due to Hashimoto’s disease may benefit more from Levothyroxine therapy [18]. On the other hand, some other studies indicate that treatments with Levothyroxine have not always successfully restored the immunoglobulin to its normal levels [9].

Hypothyroidism could be considered as T helper1 disease [21] in which pro-inflammatory cytokines such as TNF- α and IL-6 play a crucial role [22,23]. There is a connection between the level of TPO Abs and pro-inflammatory cytokines like TNF-α and IL-6 [24]. The antibodies produced against the antigens specific to the thyroid like TPO, result in an immunity complex, which activates the complement pathway and ultimately the T cells, thus leading to an increase in the production of pro-inflammatory cytokines [24]. Therefore, Anti-TPO Abs, which play a predictive role in the progress of hypothyroidism can have either direct cytotoxic effect for thyroid cells through the IgG1 class [24,25], or indirect destructive effect on thyrocytes through activating TH1 cells and increasing inflammatory responses via release of inflammatory cytokines [26]. In addition, the secretory function of monocytes and lymphocytes is associated with the TPO Abs titer which can be accounted for the fact that the interaction between the monocytes and T cells activates B lymphocytes and produces TPO Abs [18]. Monocytes, lymphocytes and cytokines produced by these cells play an essential role in triggering autoimmunity disorders [27,28]. The monocytes, macrophages, lymphocytes coupled with the cytokines secreted by these cells – in particular, TNF-α and IL-6 – contribute to insulin resistance and atherosclerotic plaques [16,29]. Studies have shown, Levothyroxine treatment increases the number of CD8+ cells and may impedes the progress of the disease [30,31]. As a result, the anti-inflammatory effects of Levothyroxine could be accompanied by a decrease in the TPO Abs titer. This implies that the reduction in the TPO Abs titer stems from a drop in the secretion of the cytokines involved in the underlying mechanism of the disease [18]. The findings of the present study show that with the exception of patients with high TPO Abs serum levels of above 1000 IU/ml, insulin resistance is uninfluenced by existence of Anti TPO antibody in hypothyroid patients being treated with Levothyroxine, a fact which might be due to the anti-inflammatory and curative effects of Levothyroxine.

Moreover, the present study shows that euthyroid subjects in the highly positive Anti TPO Abs group have lower serum HDL level than the rest of the subjects (P = 0.04). Except to the subgroup of Hashimoto’s thyroiditis with highly positive antibody levels, overall we could not find any difference between patients with and without autoimmunity regarding their lipid profile. Therefore, if we consider any association between anti thyroid antibodies and HDL-c or insulin resistance it might be true just for much higher levels of autoantibodies. There is a raft of research available concerning the effect of Levothyroxine on lipid profile which illustrates that levothyroxine can indeed help reduce total cholesterol and LDL to a limited extent [31,32]. Yet, the effect of Levothyroxine on the serum level of HDL remains unverified. Some studies maintain that levothyroxine exerts no discernible effect on HDL serum levels [18,33]) whereas others contend that it escalates HDL serum levels [32,34]. Our results were partially in line with Tamer et al. [35] who have concluded that the thyroid autoimmunity disorder can be proved to be correlated with hyperlipidemia and atherosclerosis, independent of the thyroid function. According to the study, the serum level of TPO Abs bears a negative relationship with HDL; which is confirmed in another study where a positive correlation between Anti-TPO Abs and cholesterol and LDL is also reported [36].

In conducting this research, few restrictions were encountered. The first one was the relatively limited size of the samples. Obviously, a larger number of samples are required to be able to extrapolate the results especially in a subgroup with highly positive Anti TPO Abs. Secondly, female subjects greatly outnumbered the males, thus making it impossible to generalize the results for male patients with hypothyroidism. Thirdly, all the participants had been receiving levothyroxine and were euthyroid at the start of this research. The strategy was selected in the design of this study to eliminate the effect of hypothyroxinemia itself, as a confounding factor, on the results. However, as long as it is believed that Levothyroxine treatment may cause some attenuation in the immune system’s performance the results might be affected by this and it would be more beneficial to include subjects who have been recently diagnosed with hypothyroidism and have not received any treatment yet in a separate study.

## Conclusions

The present study demonstrated that there is no obvious association between thyroid autoimmunity and metabolic indexes of hypothyroid patients. Only patients with Anti TPO antibody levels more than 1000 IU/ml may experience higher insulin level and less HDL-c with the same BMI. More studies with higher sample size of patients with highly positive antibodies would be more informative.

## Abbreviations

HT: Hashimoto’s thyroiditis
TSH: Thyroid-Stimulating Hormone
FT: Free thyroxin
Anti-TPO Abs: Anti-Thyroid Peroxidase Antibodies
IR: Insulin resistance
HOMA: Homeostasis model assessment
FBG: Fasting Blood Glucose
OGTT: Oral Glucose Tolerance Test
TC: Total cholesterol
HDL-C: High density lipoprotein-cholesterol
TG: Triglyceride
WC: Waist circumference
BMI: Body Mass Index
SBP: Systolic blood pressure
DSB: Diastolic Blood Pressure
